# Supplementary material for: Plant hairy roots for the production of extracellular vesicles with antitumor bioactivity
Source: Commun Biol. 2022 Aug 20;5:848. doi: 10.1038/s42003-022-03781-3 (PMC9392725; doi:10.1038/s42003-022-03781-3)
Supplement: Supplementary file 2 — Supplementary Information [file 42003_2022_3781_MOESM2_ESM.pdf]

Plant hairy roots for the production of extracellular vesicles with antitumor bioactivity

Eleonora Boccia[1#], Mariaevelina Alfieri[1,2#], Raffaella Belvedere[1], Valentina Santoro[1], Marianna Colella, Pasquale Del Gaudio[1], Maria Moros[3,4], Fabrizio Dal Piaz[5,6], Antonello Petrella[1], Antonietta Leone[1], Alfredo Ambrosone[1*]

Affiliations

[1] Department of Pharmacy, University of Salerno, 84084, Fisciano, Italy;

[2] Clinical Pathology, Pausilipon Hospital, A.O.R.N Santobono-Pausilipon, 80123, Naples, Italy

[3] Instituto de Nanociencia y Materiales de Aragón (INMA), CSIC-Universidad de Zaragoza, Zaragoza, Spain

[4] Biomedical Research Networking Center in Bioengineering, Biomaterials and Nanomedicine (CIBER-BBN), Spain

[5] Department of Medicine, Surgery and Dentistry "Scuola Medica Salernitana", University of Salerno, 84081 Baronissi, Italy

[6] Operative Unit of Clinical Pharmacology, University Hospital "San Giovanni di Dio e Ruggi d'Aragona", 84131 Salerno, Italy

[#] these authors contributed equally

***Correspondence:** aambrosone@unisa.it;

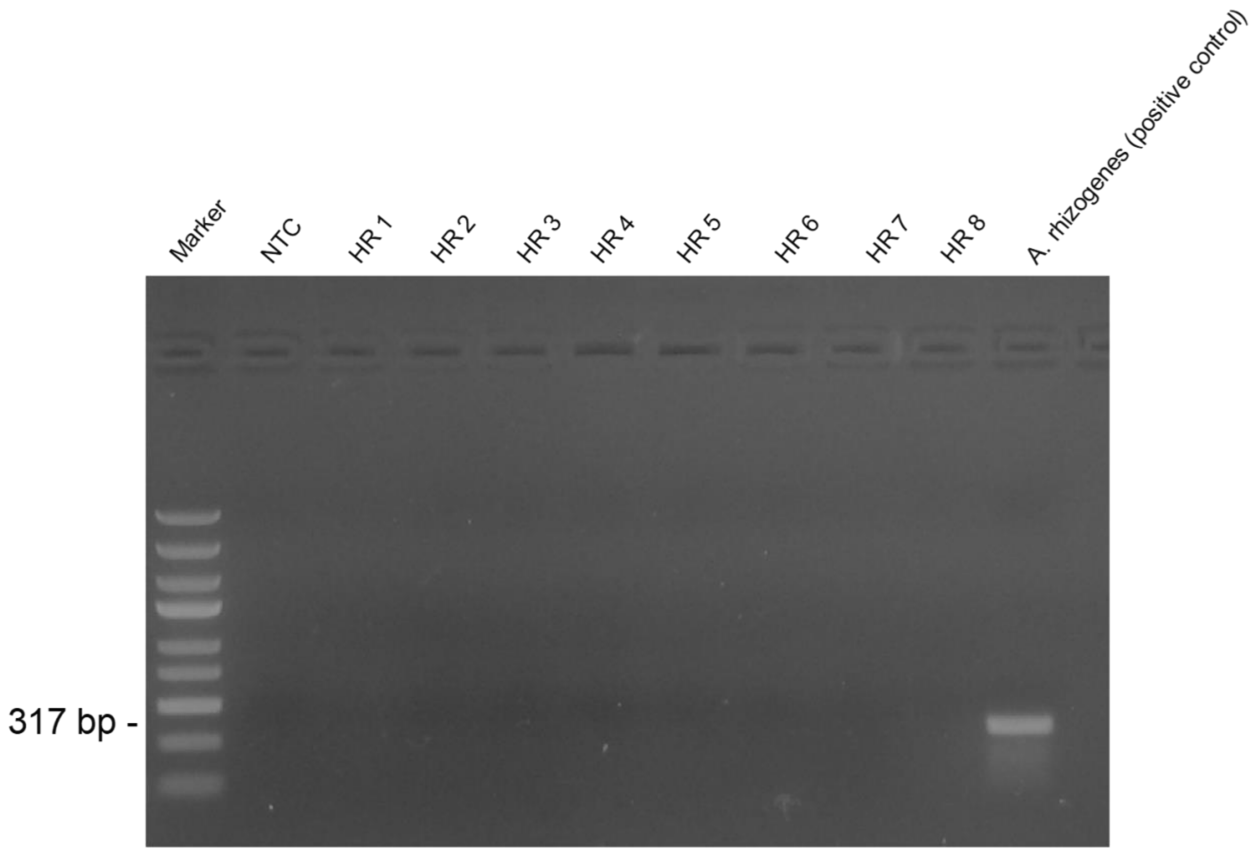

Figure S1. *VirD2* amplification by PCR in independent hairy root lines (HR). Genomic DNA from *Agrobacterium rhizogenes* was used as a positive control

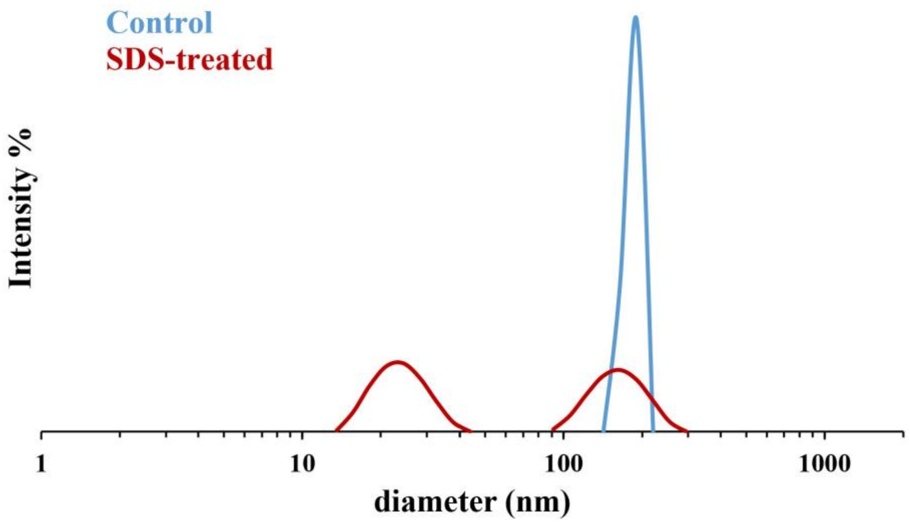

Figure S2. Particle-size distributions measured using dynamic light scattering in HR-derived EVs treated with 1%SDS (red line) and in native EV preparations (blue line).

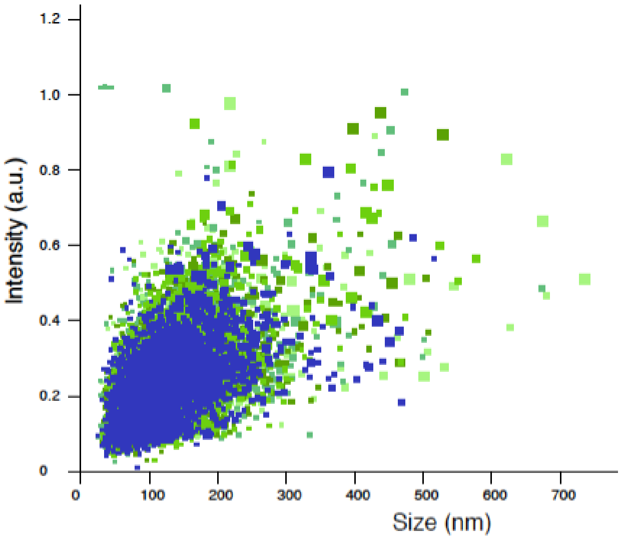

Figure S3. **EV size distribution by NTA intensity measurements**. Colours (from green to blue) represent size distribution of five independent measurements.

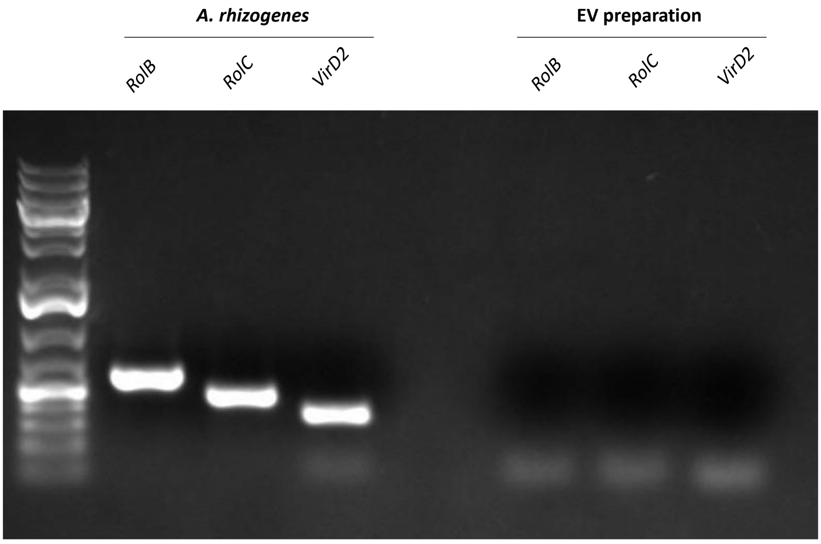

Figure S4. Absence of *rolB*, *rolC* and *virD2* genes in EV preparation checked by PCR. As positive control *A. rhizogenes* DNA has been used.

Table S1. Proteins identified only in *S. dominica* HR-derived EV by proteomic analyses performed on two independent experiments

Accession	Description
G3PC_DIACA	Glyceraldehyde-3-phosphate dehydrogenase, cytosolic OS=Dianthus caryophyllus
G3PC2_ORYSJ	Glyceraldehyde-3-phosphate dehydrogenase 2, cytosolic OS=Oryza sativa subsp. japonica
EF1A1_DAUCA	Elongation factor 1-alpha OS=Daucus carota
HSP70_SOYBN	Heat shock 70 kDa protein OS=Glycine max
METE1_ORYSJ	5-methyltetrahydropteroyltriglutamate--homocysteine methyltransferase 1 OS=Oryza sativa subsp. japonica
BIP1_ARATH	Heat shock 70 kDa protein BIP1 OS=Arabidopsis thaliana
ALF2_PEA	Fructose-bisphosphate aldolase, cytoplasmic isozyme 2 OS=Pisum sativum
METE3_ARATH	5-methyltetrahydropteroyltriglutamate--homocysteine methyltransferase 3, chloroplastic OS=Arabidopsis th
API3_SOLTU	Aspartic protease inhibitor 3 (Fragment) OS=Solanum tuberosum
PGKH2_ARATH	Phosphoglycerate kinase 2, chloroplastic OS=Arabidopsis thaliana
1433B_TOBAC	14-3-3-like protein B OS=Nicotiana tabacum
14337_ARATH	14-3-3-like protein GF14 nu OS=Arabidopsis thaliana
CDC48_SOYBN	Cell division cycle protein 48 homolog OS=Glycine max
ATPAM_SOYBN	ATP synthase subunit alpha, mitochondrial OS=Glycine max
LOX1_LENCU	Linoleate 9S-lipoxygenase OS=Lens culinaris
14331_SOLTU	14-3-3-like protein OS=Solanum tuberosum
MDHM_CITLA	Malate dehydrogenase, mitochondrial OS=Citrullus lanatus
ENPL_CATRO	Endoplasmin homolog OS=Catharanthus roseus
VCL1_PEA	Vicilin, 14 kDa component OS=Pisum sativum
ENO2_ARATH	Bifunctional enolase 2/transcriptional activator OS=Arabidopsis thaliana
14339_SOLLC	14-3-3 protein 9 OS=Solanum lycopersicum
PHSL_VICFA	Alpha-1,4 glucan phosphorylase L isozyme, chloroplastic/amyloplastic OS=Vicia faba
FRI_PHAVU	Ferritin, chloroplastic OS=Phaseolus vulgaris
RS31_ARATH	40S ribosomal protein S3-1 OS=Arabidopsis thaliana
RS8_MAIZE	40S ribosomal protein S8 OS=Zea mays
RAA2C_ARATH	Ras-related protein RABA2c OS=Arabidopsis thaliana
RSSA_DAUCA	40S ribosomal protein SA OS=Daucus carota
MANA1_ARATH	Alpha-mannosidase At3g26720 OS=Arabidopsis thaliana
RS6_ASPOF	40S ribosomal protein S6 OS=Asparagus officinalis
IF4A1_ARATH	Eukaryotic initiation factor 4A-1 OS=Arabidopsis thaliana
HSP80_SOLLC	Heat shock cognate protein 80 OS=Solanum lycopersicum
RGP1_ARATH	UDP-arabinopyranose mutase 1 OS=Arabidopsis thaliana
NDK1_SOYBN	Nucleoside diphosphate kinase 1 OS=Glycine max
MANA2_ARATH	Probable alpha-mannosidase At5g13980 OS=Arabidopsis thaliana
GLYC4_ARATH	Serine hydroxymethyltransferase 4 OS=Arabidopsis thaliana
PGKY_WHEAT	Phosphoglycerate kinase, cytosolic OS=Triticum aestivum
TCTP_MAIZE	Translationally-controlled tumor protein homolog OS=Zea mays
RL12_PRUAR	60S ribosomal protein L12 OS=Prunus armeniaca
SCRK_SOLTU	Fructokinase OS=Solanum tuberosum
RL11_MEDSA	60S ribosomal protein L11 OS=Medicago sativa

DPYS_ARATH	Dihydropyrimidinase OS=Arabidopsis thaliana
RAE1E_ARATH	Ras-related protein RABE1e OS=Arabidopsis thaliana
CALR_MAIZE	Calreticulin OS=Zea mays
AB43G_ORYSJ	ABC transporter G family member 43 OS=Oryza sativa subsp. japonica
NLTP_DAUCA	Non-specific lipid-transfer protein OS=Daucus carota
MANA_CANEN	Alpha-mannosidase OS=Canavalia ensiformis
REHY_MEDTR	1-Cys peroxiredoxin OS=Medicago truncatula
RS4_GOSHI	40S ribosomal protein S4 OS=Gossypium hirsutum
FTHS_SPIOL	Formate--tetrahydrofolate ligase OS=Spinacia oleracea
UXS3_ARATH	UDP-glucuronic acid decarboxylase 3 OS=Arabidopsis thaliana
NLTP1_VIGRR	Non-specific lipid-transfer protein 1 OS=Vigna radiata var. radiata
RS30_ARATH	40S ribosomal protein S30 OS=Arabidopsis thaliana
CALR_PRUAR	Calreticulin OS=Prunus armeniaca
RS254_ARATH	40S ribosomal protein S25-4 OS=Arabidopsis thaliana
MDHC_MAIZE	Malate dehydrogenase, cytoplasmic OS=Zea mays
RB11A_TOBAC	Ras-related protein Rab11A OS=Nicotiana tabacum
RL73_ARATH	60S ribosomal protein L7-3 OS=Arabidopsis thaliana
UGDH3_ARATH	UDP-glucose 6-dehydrogenase 3 OS=Arabidopsis thaliana
YPTV2_VOLCA	GTP-binding protein yptV2 OS=Volvox carteri
H4_ARATH	Histone H4 OS=Arabidopsis thaliana
BADH1_ARATH	Aminoaldehyde dehydrogenase ALDH10A8, chloroplastic OS=Arabidopsis thaliana
OBP2B_MAIZE	Oil body-associated protein 2B OS=Zea mays
RL182_ARATH	60S ribosomal protein L18-2 OS=Arabidopsis thaliana
AADH2_PEA	Aminoaldehyde dehydrogenase 2, peroxisomal OS=Pisum sativum
RLA0_SOYBN	60S acidic ribosomal protein P0 OS=Glycine max
LEGB_PEA	Legumin B (Fragment) OS=Pisum sativum
SODC5_MAIZE	Superoxide dismutase [Cu-Zn] 4AP OS=Zea mays
RGP1_PHODC	Probable UDP-arabinopyranose mutase 1 (Fragments) OS=Phoenix dactylifera
ACO3M_ARATH	Aconitate hydratase 3, mitochondrial OS=Arabidopsis thaliana
RS12_HORVU	40S ribosomal protein S12 OS=Hordeum vulgare
LOX4_SOYBN	Linoleate 9S-lipoxygenase-4 OS=Glycine max
SUS_SOYBN	Sucrose synthase OS=Glycine max OX=3847 GN=SS PE=1 SV=
RS19_ORYSJ	40S ribosomal protein S19 OS=Oryza sativa subsp. japonica
G6PI1_CLAFR	Glucose-6-phosphate isomerase, cytosolic 1 OS=Clarkia franciscana
RL40A_ARATH	Ubiquitin-60S ribosomal protein L40-1 OS=Arabidopsis thaliana
TENAE_SOYBN	Probable bifunctional TENA-E protein OS=Glycine max
REHY_MAIZE	1-Cys peroxiredoxin PER1 OS=Zea mays
RAA4A_ARATH	Ras-related protein RABA4a OS=Arabidopsis thaliana
SNAK1_SOLTU	Snakin-1 OS=Solanum tuberosum
FTHS_ARATH	Formate--tetrahydrofolate ligase OS=Arabidopsis thaliana
RL9_PEA	60S ribosomal protein L9 OS=Pisum sativum
RS281_ARATH	40S ribosomal protein S28-1 OS=Arabidopsis thaliana
CP18C_ARATH	Peptidyl-prolyl cis-trans isomerase CYP18-3 OS=Arabidopsis thaliana
RS16_FRIAG	40S ribosomal protein S16 OS=Fritillaria agrestis
RS13_SOYBN	40S ribosomal protein S13 OS=Glycine max
TET7_ARATH	Tetraspanin-7 OS=Arabidopsis thaliana

R10A1_ARATH	60S ribosomal protein L10a-1 OS=Arabidopsis thaliana
MASY_SOYBN	Malate synthase, glyoxysomal (Fragment) OS=Glycine max
PDIA6_MEDSA	Probable protein disulfide-isomerase A6 OS=Medicago sativa
ANX4_FRAAN	Annexin-like protein RJ4 OS=Fragaria ananassa
TBB3_SOYBN	Tubulin beta chain (Fragment) OS=Glycine max
TIP31_ARATH	Aquaporin TIP3-1 OS=Arabidopsis thaliana
GBLP_SOYBN	Guanine nucleotide-binding protein subunit beta-like protein OS=Glycine max
CONA1_LUPAN	Conglutin alpha 1 OS=Lupinus angustifolius
MAOX_POPTR	NADP-dependent malic enzyme OS=Populus trichocarpa
ACBP5_ARATH	Acyl-CoA-binding domain-containing protein 5 OS=Arabidopsis thaliana
RS141_MAIZE	40S ribosomal protein S14 OS=Zea mays
SODM_HEVBR	Superoxide dismutase [Mn], mitochondrial OS=Hevea brasiliensis
GDI_ARATH	Guanosine nucleotide diphosphate dissociation inhibitor At5g09550 OS=Arabidopsis thaliana
PGMP_PEA	Phosphoglucomutase, chloroplastic OS=Pisum sativum
COPDA_ARATH	Probable cytosolic oligopeptidase A OS=Arabidopsis thaliana
IF5A1_SOLTU	Eukaryotic translation initiation factor 5A-1/2 OS=Solanum tuberosum
GLYG1_SOYBN	Glycinin G1 OS=Glycine max
ALL11_ARAHY	Allergen Ara h 1, clone P17 OS=Arachis hypogaea
ALL12_ARAHY	Allergen Ara h 1, clone P41B OS=Arachis hypogaea
GLCA1_SOYBN	Beta-conglycinin alpha subunit 1 OS=Glycine max
H32_CICIN	Histone H3.2 OS=Cichorium intybus
EBF1_ARATH	EIN3-binding F-box protein 1 OS=Arabidopsis thaliana
RGA3_SOLBU	Putative disease resistance protein RGA3 OS=Solanum bulbocastanum
RLM1B_ARATH	Disease resistance protein RML1B OS=Arabidopsis thaliana
PERN1_TOBAC	Peroxidase N1 OS=Nicotiana tabacum
G3PC2_HORVU	Glyceraldehyde-3-phosphate dehydrogenase 2, cytosolic (Fragment) OS=Hordeum vulgare
GRDH_DAUCA	Glucose and ribitol dehydrogenase OS=Daucus carota
SUVH7_ARATH	Histone-lysine N-methyltransferase, H3 lysine-9 specific SUVH7 OS=Arabidopsis thaliana
GDL77_ARATH	GDSL esterase/lipase At5g18430 OS=Arabidopsis thaliana
TB1_ORYSJ	Transcription factor TB1 OS=Oryza sativa subsp. japonica
BXL4_ARATH	Beta-D-xylosidase 4 OS=Arabidopsis thaliana
TAF1_ARATH	Transcription initiation factor TFIID subunit 1 OS=Arabidopsis thaliana
BBE14_ARATH	Berberine bridge enzyme-like 14 OS=Arabidopsis thaliana
EGY2_ARATH	Probable zinc metalloprotease EGY2, chloroplastic OS=Arabidopsis thaliana
ACT_ACEPE	Actin (Fragment) OS=Acetabularia peniculus
CRS1_MAIZE	Chloroplastic group IIA intron splicing facilitator CRS1, chloroplastic OS=Zea mays
STSYN_PEA	Stachyose synthase OS=Pisum sativum
GLYG4_SOYBN	Glycinin G4 OS=Glycine max
GLUA1_ORYSJ	Glutelin type-A 1 OS=Oryza sativa subsp. japonica
KN5B_ARATH	Kinesin-like protein KIN-5B OS=Arabidopsis thaliana
CDPK8_ORYSJ	Calcium-dependent protein kinase 8 OS=Oryza sativa subsp. japonica
RCA_CHLRE	Ribulose bisphosphate carboxylase/oxygenase activase, chloroplastic OS=Chlamydomonas reinhardtii
SSG1_ORYGL	Granule-bound starch synthase 1, chloroplastic/amyloplastic OS=Oryza glaberrima
NUA_ARATH	Nuclear-pore anchor OS=Arabidopsis thaliana
GLYG2_SOYBN	Glycinin G2 OS=Glycine max
ALA8_ARATH	Probable phospholipid-transporting ATPase 8 OS=Arabidopsis thaliana

POD1_ARATH	Protein POLLEN DEFECTIVE IN GUIDANCE 1 OS=Arabidopsis thaliana
Y1143_ARATH	Probable LRR receptor-like serine/threonine-protein kinase At1g14390 OS=Arabidopsis thaliana
FTSI1_ARATH	Probable inactive ATP-dependent zinc metalloprotease FTSHI 1, chloroplastic OS=Arabidopsis thaliana
CFM9_ARATH	CRM-domain containing factor CFM9, mitochondrial OS=Arabidopsis thaliana
TI10A_ARATH	Protein TIFY 10A OS=Arabidopsis thaliana
NORK_MEDTR	Nodulation receptor kinase OS=Medicago truncatula
PP306_ARATH	Pentatricopeptide repeat-containing protein At4g11690 OS=Arabidopsis thaliana
MAOP1_ARATH	NADP-dependent malic enzyme 1 OS=Arabidopsis thaliana
FDM1_ARATH	Factor of DNA methylation 1 OS=Arabidopsis thaliana
WBC30_ARATH	Putative white-brown complex homolog protein 30 OS=Arabidopsis thaliana
HDG9_ARATH	Homeobox-leucine zipper protein HDG9 OS=Arabidopsis thaliana

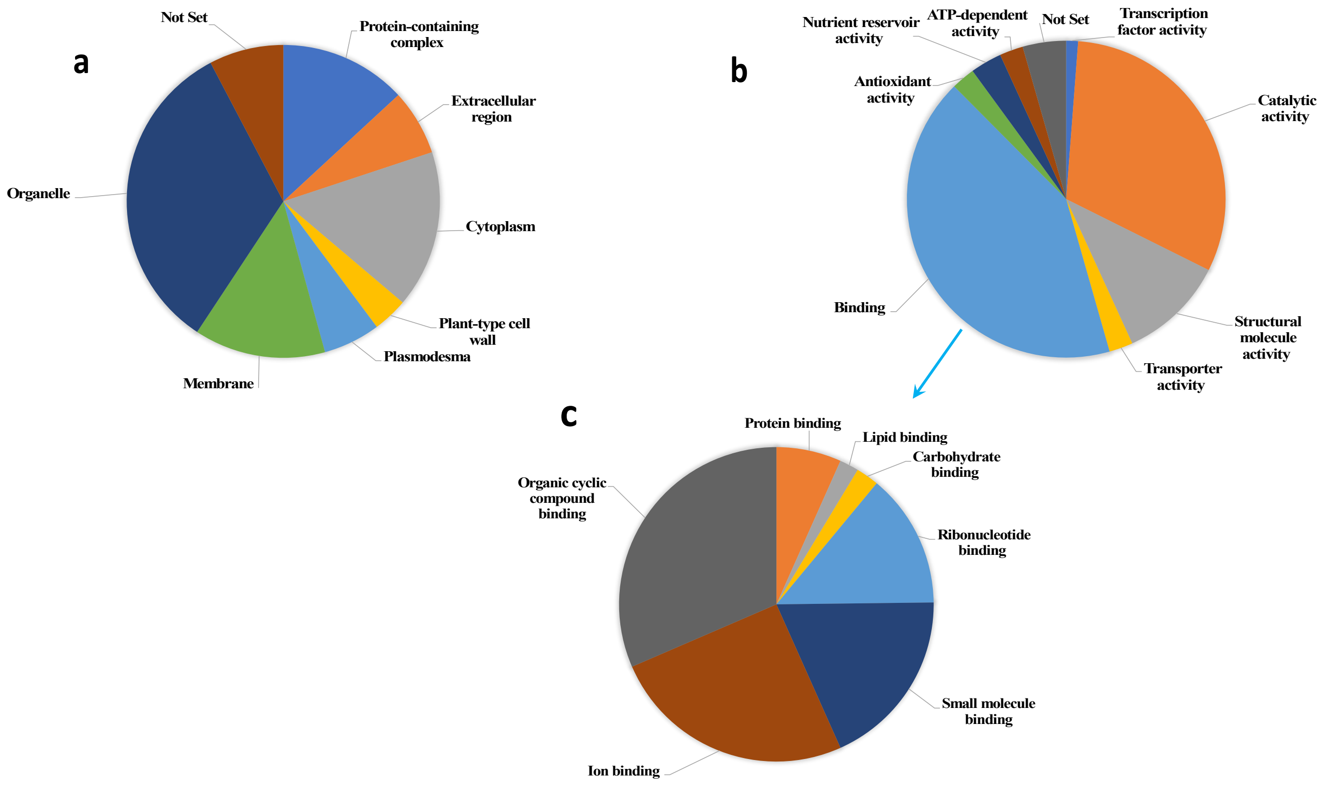

Figure S5. Distribution of Gene Ontology annotation of proteins selectively identified in HR-derived EV preparations. **a** Cell components analysis; **b** and **c** Molecular function analysis.

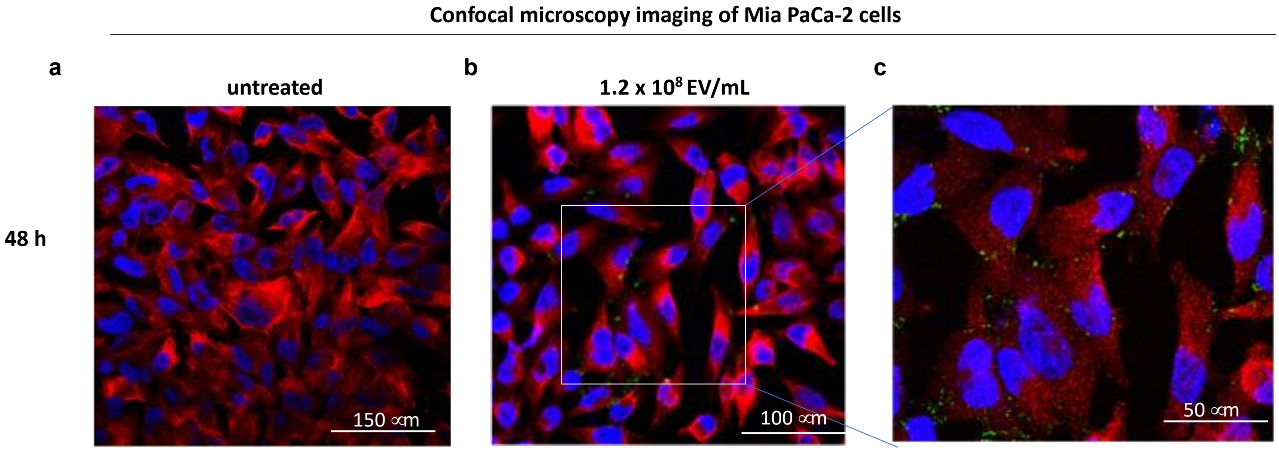

Figure S6. EV uptake in MIA PaCa-2 cells after 48 h incubation. **a** untreated MIA PaCa-2 cells. **b** MIA PaCa-2 cells treated with Fluo-EVs for 48 h. **c** Close up view of b. Scale bars: 150 µm in a and b, 50 µm in c.

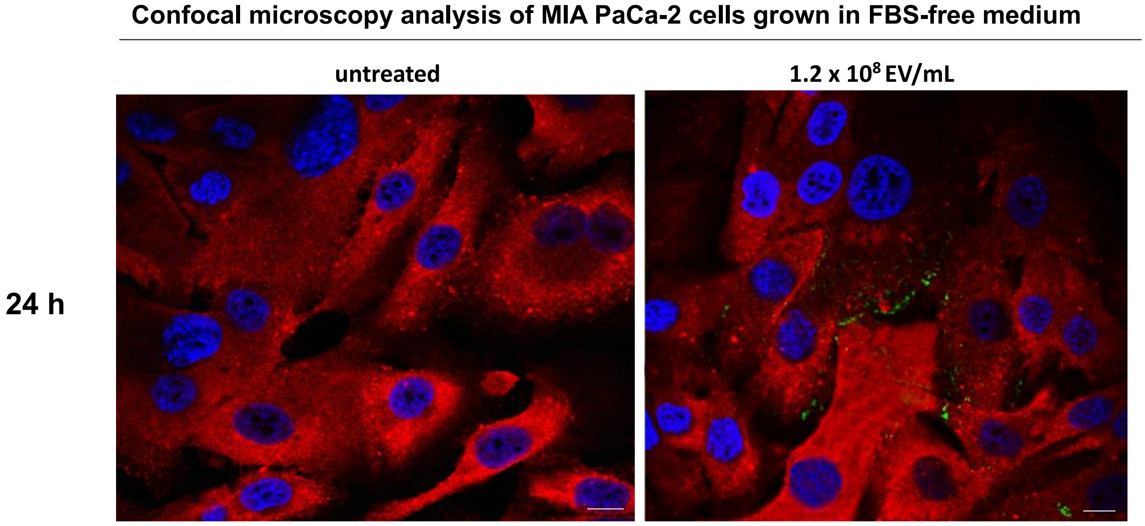

Figure S7. HR-derived EV uptake in MIA PaCa-2 cells after 24 h incubation in a Fetal Bovine Serum-free medium. Scale bars: 50µm.

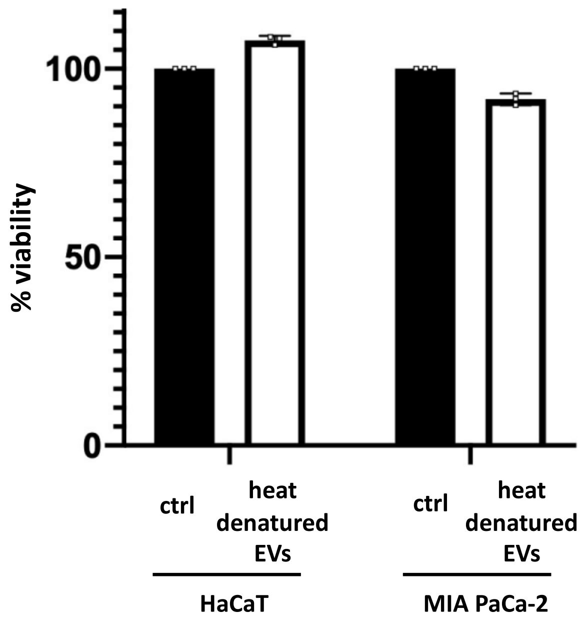

Figure S8. Effects of heat-denatured HR-derived EVs (3 x 10^8 particle/mL) after 24 h incubation in human keratinocytes and MIA PaCa-2 cancer cells. The values were presented as means ± SD of three independent experiments carried out in triplicate.

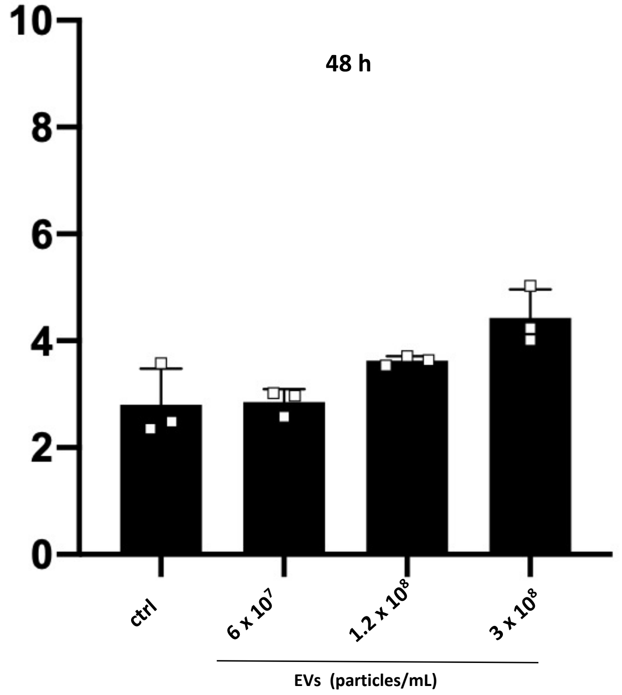

Figure S9. Apoptotic cell counts by cytofluorimetric assay in EVs-treated HaCat cells upon 48 h exposure. The values reported in the graphs are the mean ± SD from 3 independent experiments performed in technical triplicates.

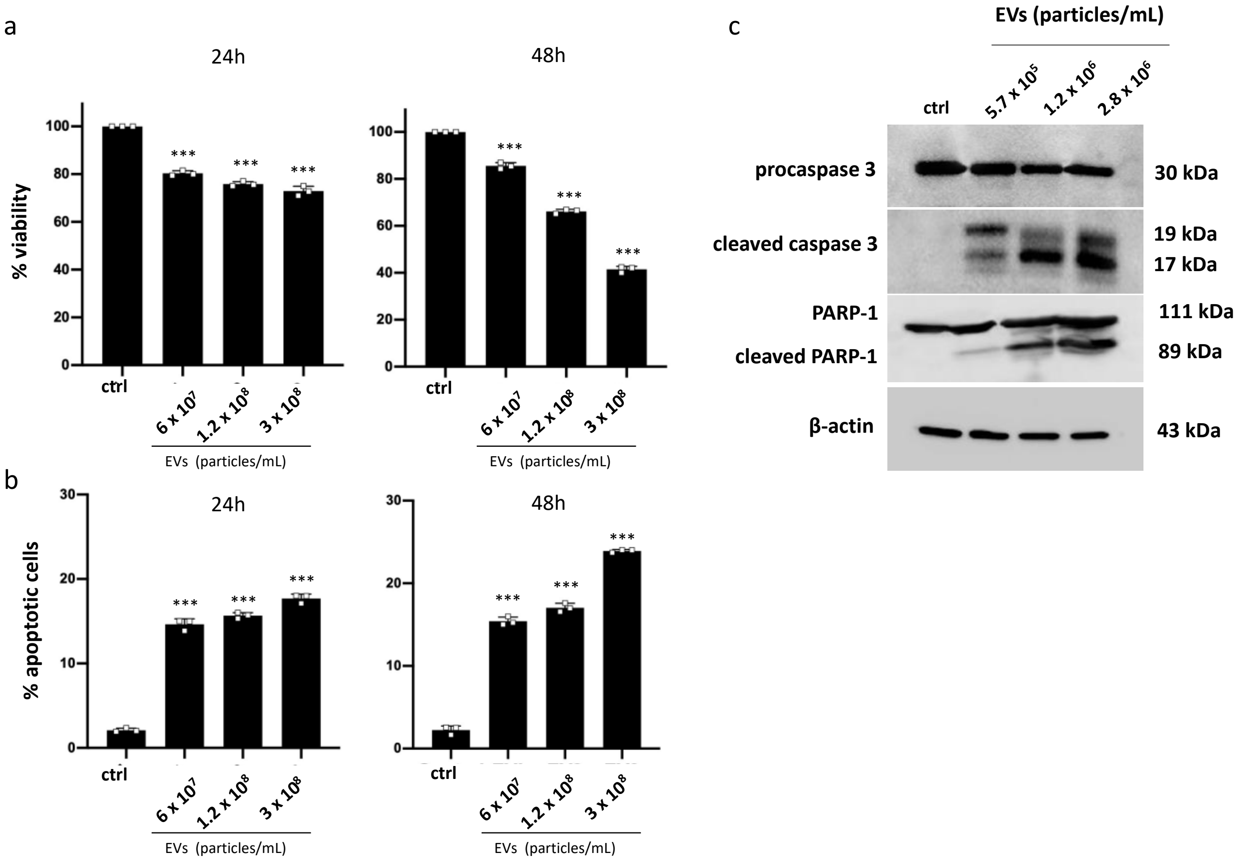

Figure S10. Effects of HR-derived EVs after 24 h and 48 h incubation in MCF-7 breast cancer cells. **a** MTT colorimetric assay on MCF-7 cells after 24 h (left) and 48 h (right) of EV treatments, respectively. **b** Analyses of apoptotic cells by cytofluorimetric assay in EVs-treated MCF-7 cells upon 24 h and 48 h exposure (left and right bar chart, respectively). The values reported in the graphs are the mean ± SD from at least 3 independent experiments performed in technical triplicates. The asterisks denote significant differences between treatments and untreated controls (* p<0.05; ** p<0.01; *** p<0.001) according to Student's t test. **c** Western blot analyses of protein extracts from MCF-7 cells treated for 24 h with different EV concentrations.

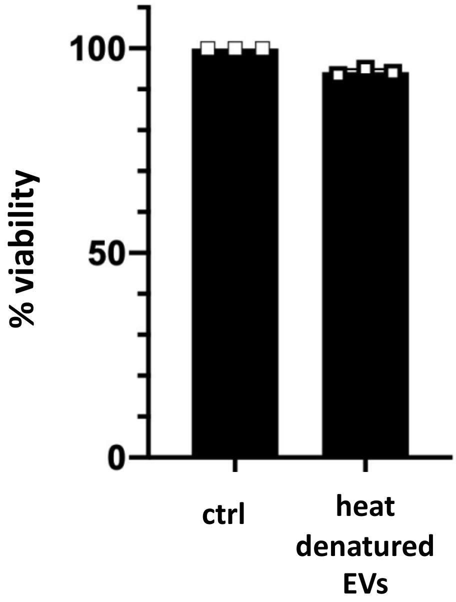

Figure S11. Effects of heat-denatured HR-released EVs (3 x 10^8 particle/mL) on MCF-7 viability after 24 h incubation. The values were presented as means ± SD of three independent experiments carried out in triplicate

procaspase 3 in MIA PaCa-2 cells

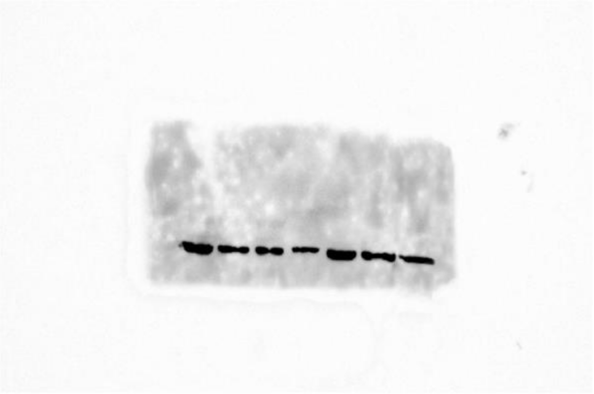

PARP-1 in MIA PaCa-2 cells

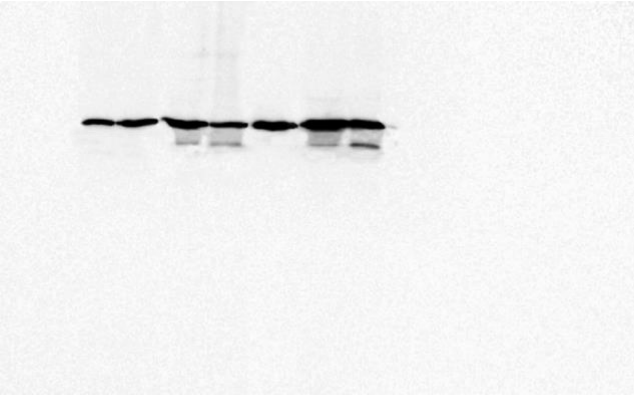

Cleaved caspase 3 in MIA PaCa-2 cells

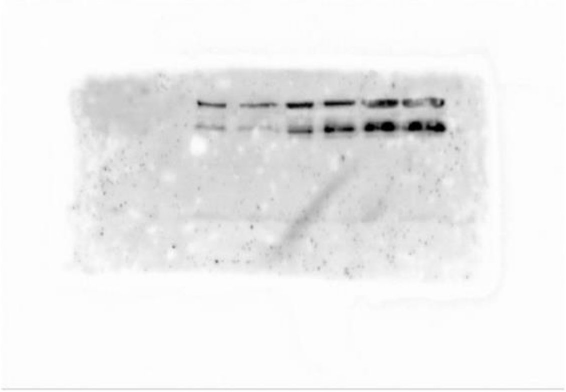

β-actin in MIA PaCa-2

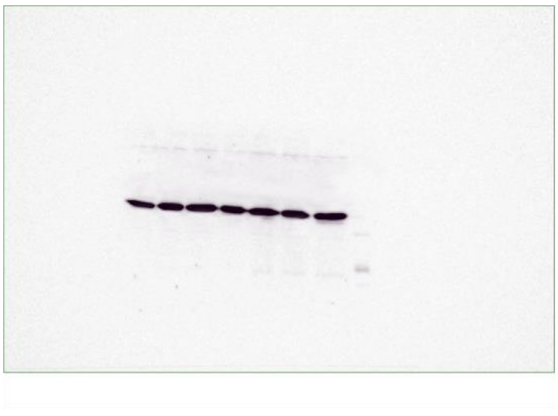

Figure S12. Uncropped images of western blots reported in figure 3.

procaspase 3 in MCF7 cells

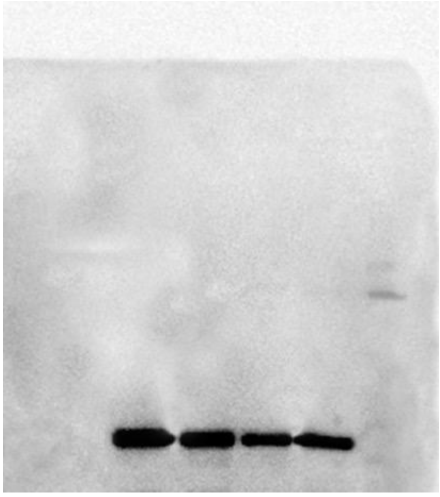

PARP-1 in MCF7 cells

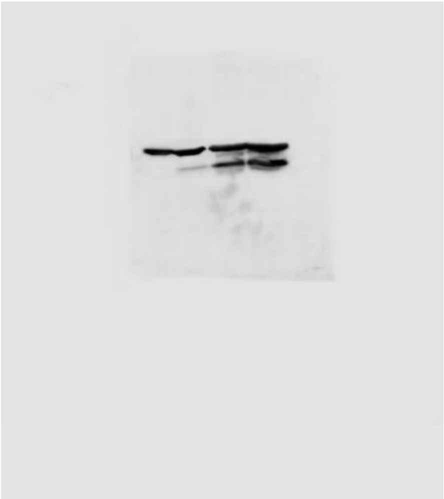

cleaved caspase 3 MFF7 cells

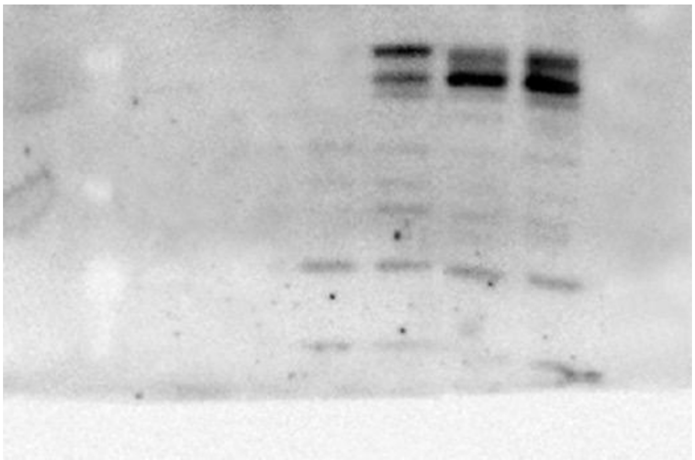

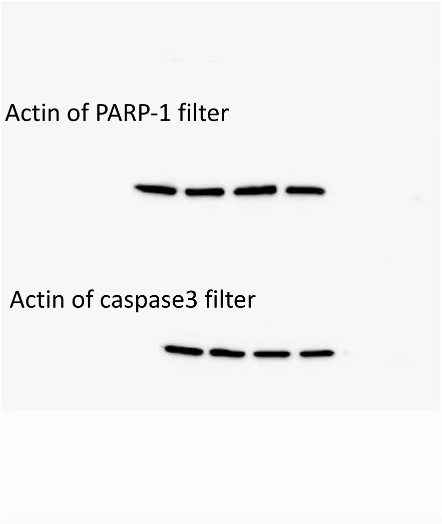

Figure S13. Uncropped images of western blots reported in figure S10.
