# Supplementary material for: Plant hairy roots for the production of extracellular vesicles with antitumor bioactivity
Source: Commun Biol. 2022 Aug 20;5:848. doi: 10.1038/s42003-022-03781-3 (PMC9392725; doi:10.1038/s42003-022-03781-3)
Supplement: Supplementary file 3 — Description of Additional Supplementary Files [file 42003_2022_3781_MOESM3_ESM.pdf]

Description of Additional Supplementary Files

File name: Supplementary Data 1
Description: The list of proteins commonly found in the EV preparations and in the nonvesicular fractions.

File name: Supplementary Data 2
Description: Raw data of biological assays reported in the main figures
